# The Beginning of the End

**Published:** 1891-08-01

**Authors:** 


### Passing Topics.

### THE BEGINNING OF THE END.

The Lords' Select Committee which called Colonel Montefiore as its earliest witness, fitly and appropriately summoned Mr. C. S. Loch as its last, and thus a degree of form and symmetry, if not of harmony, is preserved to the proceedings, and whatever variations may have taken place during the progress of the performance, we are now brought back in orthodox Rondo fashion to the original air, and are reminded that its alpha and omega proceed from a common inspiration, though they are pitched, as it would seem, in somewhat different keys.

It is of course well known that at the outset there was something in the nature of an indictment of our hospitals, and although we must not attempt to forestall the conclusions of the Committee in any important particular, we may say, without impropriety, that a great deal of the evidence given must have proved far from pleasant reading for the party who had hoped to convict our great medical institutions of all sorts of shortcomings and excesses.

We congratulate Mr. Loch that he has had the good sense to learn something from the evidence, which we have his word he has followed "more or less closely," and the result is that he appears to have softened some of those harsher criticisms with the application of which the Charity Organization Society has been always credited. Indeed, not a few of the opinions and expressions he made use of are emphatically appreciative, and it says much for the way in which the hospitals generally have passed through the ordeal of examination, that the tone now adopted towards them is distinctly less carping and hostile.

Earl Spencer has already announced that the administration of the hospitals has favourably impressed him, and, we suppose, his colleagues, and we are in no fear that the formal report will fail to do justice to those meritorious institutions. Similarly, apparently, the Charity Organization Society sees reason to modify some of its earlier utterances. At any rate, instead of the wholesale and unmeasured condemnation we were led to look for, we find the society's representative officer offering reasonable and temperate suggestions, and even hesitating to assert that medical relief in London is more than sufficient for the wants of the population.

That is to say, the long continued enquiries of the Committee have failed, in the opinion of Mr. Loch, to establish the chief charge alleged against the existing method of hospital relief. On our part, we are quite ready to agree with the suggestion that "it is questionable whether releif is adjusted so as to be most suitable to the needs of the people," but we must express surprise at the contention that medical and other forms of relief are equal in respect of their pauperising tendency. Surely, no great depth of observation is requisite to discover that the granting of medical relief to a sick man may be a potent means of saving him from pauperisation, and, consequentially, it is a legitimate if by no means a novel proposition that the tendency of our hospitals is the very reverse of pauperising. Like other good things, philanthropic medical relief may be used, or it may be abused. We shall trust to the Lords' Committee to avoid drawing general conclusions from individual delinquencies.

Agricola.

